# Hyperoside protects against oxidative stress-mediated photoreceptor degeneration: therapeutic potentials for photoreceptor degenerative diseases

**DOI:** 10.1186/s12967-023-04459-y

**Published:** 2023-08-24

**Authors:** Daijin Li, Jing Xu, Jie Chang, Yujue Wang, Xiaoye Du, Hanhan Wu, Jingang Cui, Peiwei Wang, Teng Zhang, Yu Chen

**Affiliations:** 1 Yueyang Hospital of Integrated Traditional Chinese and Western Medicine, Shanghai University of Traditional Chinese Medicine, Shanghai, 200437 China; 2 Clinical Research Institute of Integrative Medicine, Shanghai Academy of Traditional Chinese Medicine, Shanghai, 200437 China; 3 Laboratory of Clinical and Molecular Pharmacology, Yueyang Hospital of Integrated Traditional Chinese and Western Medicine, Shanghai University of Traditional Chinese Medicine, Shanghai, 200437 China

**Keywords:** Hyperoside, Oxidative stress, Cell death, Photoreceptor degeneration, Retinal homeostasis

## Abstract

**Background:**

Photoreceptor degeneration underpinned by oxidative stress-mediated mitochondrial dysfunction and cell death leads to progressive and irreversible vision impairment. Drug treatments that protect against photoreceptor degeneration are currently available in the clinical settings. It has been shown that hyperoside, a flavonol glycoside, protects against neuronal loss in part by suppressing oxidative stress and maintaining the functional integrity of mitochondria. However, whether hyperoside protects against photoreceptor degeneration remains unknown.

**Methods:**

To address the pharmacological potentials of hyperoside against oxidative stress-mediated photoreceptor degeneration on molecular, cellular, structural and functional levels, multiple in vitro and in vivo methodologies were employed in the current study, including live-cell imaging, optical coherence tomography, electroretinography, histological/immunohistochemical examinations, transmission electron microscopy, RNA-sequencing and real-time qPCR.

**Results:**

The in vitro results demonstrate that hyperoside suppresses oxidative stress-mediated photoreceptor cell death in part by mitigating mitochondrial dysfunction. The in vivo results reveal that hyperoside protects against photooxidative stress-induced photoreceptor morphological, functional and ultrastructural degeneration. Meanwhile, hyperoside treatment offsets the deleterious impact of photooxidative stress on multiple molecular pathways implicated in the pathogenesis of photoreceptor degeneration. Lastly, hyperoside attenuates photoreceptor degeneration-associated microglial inflammatory activation and reactive Müller cell gliosis.

**Conclusions:**

All things considered, the present study demonstrates for the first time that hyperoside attenuates oxidative stress-induced photoreceptor mitochondrial dysfunction and cell death. The photoreceptor-intrinsic protective effects of hyperoside are corroborated by hyperoside-conferred protection against photooxidative stress-mediated photoreceptor degeneration and perturbation in retinal homeostasis, warranting further evaluation of hyperoside as a photoreceptor protective agent for the treatment of related photoreceptor degenerative diseases.

## Background

The retina consists of multiple orders of neurons that collaboratively conduct light-sensing and vision-forming functions. Photoreceptors are the first-order retinal neurons carrying out specialized light-sensing and phototransduction functions, thereby contributing in an indispensable manner to the initiation of the visual processes [1]. Regardless of the etiologies, genetic or environmental, the progressive loss of photoreceptors by cell death is held responsible for irreversible vision impairment or blindness in patients with photoreceptor degenerative disorders, to name a few, dry age-related macular degeneration, Stargardt disease and retinitis pigmentosa [2]. Developing mechanisms-based pharmacological agents with photoreceptor protective effects is required given that drug treatments that protect against photoreceptor degeneration are currently available in the clinical settings [3].

The general consensus of the mechanisms underpinning photoreceptor cell death irrespective of the etiologies centers on the heightened level of oxidative stress [2]. Oxidative stress, by exerting a broad impact on multiple molecular targets and cellular processes, triggers a cascade of harmful events that invariably lead to photoreceptor cell death. With respect to photoreceptor degeneration, mitochondrial dysfunction plays a protagonist role in oxidative stress as well as oxidative stress-induced cell death, emerging as an important target of photoreceptor protective therapies [4–6]. Therefore, pharmacological agents with antioxidant and mitochondrial protective capacities are under close examination for their therapeutic potentials in attenuating photoreceptor degeneration. Natural products, rich in antioxidants, serve as a value resource for this purpose [7].

Our previous study has demonstrated that *Cuscuta chinensis* Lam., an herbal medicinal that is conventionally used for the treatment of vision impairment in traditional Chinese medicine, is effective at protecting against oxidative stress-mediated photoreceptor degeneration [8]. Hyperoside, a flavonol glycoside and the signature chemical constituent of *Cuscuta chinensis* Lam, is the most abundant flavonoids present in *Cuscuta chinensis* Lam [8]. Studies performed in experimental models of neurogenerative disorders, for instance, Alzheimer’s disease, have demonstrated that hyperoside protects against neuronal loss in part through suppressing oxidative stress and maintaining the functional integrity of mitochondria [9]. However, whether hyperoside has a direct impact on oxidative stress-induced mitochondrial dysfunction and photoreceptor cell death remains unknown.

Therefore, we set out to test the hypothesis that hyperoside may protect against photoreceptor degeneration by suppressing oxidative stress-induced mitochondrial impairment and photoreceptor cell death. Meanwhile, it is also worth noting that perturbation of the homeostasis in the retinal microenvironment occurs not only as a consequence of photoreceptor degeneration, but also plays an active role in further exacerbating photoreceptor loss and promoting the functional deterioration of the retina. The most typical changes characterizing a pathologically imbalanced retinal microenvironment include inflammatory activation of the retinal resident immune cells, microglia, as well as the gliotic responses of Müller glia, a type of macroglia uniquely present in the retina [10, 11]. Thus, to better understand the pharmacological implications of hyperoside in protecting against photoreceptor degeneration, we also assessed the impact of hyperoside on photoreceptor degeneration-associated reactive phenotypes in microglia and Müller cells in the retina.

## Methods

### Reagents

Hyperoside (purity > 98%, Lot. No. P14A11F121347) was ordered from Shanghai Yuanye Biotechnology Co., Ltd (China). Sodium nitroprusside (SNP), hydrocortisone 21-hemisuccinate, progesterone and putrescine were purchased from Sigma-Aldrich (USA). Dulbecco’s modified Eagle’s medium (DMEM) and penicillin/streptomycin were ordered from Thermo Fisher Scientific (USA). Fetal bovine serum (FBS) was purchased from Nobimpex (German).

### Cell culture and treatments

661W cone photoreceptor cells, originally obtained from Dr. Muayyad R. AI-Ubaidi [12], were cultured at 37 °C with 5% CO_2_ in DMEM supplemented with 10% FBS, 1% penicillin/streptomycin, 40 ng/mL hydrocortisone 21-hemisuccinate, 40 ng/mL progesterone and 100 μmol/L putrescine. For the experiments involving SNP stimulation, 661W cells were treated with hyperoside at the indicated concentrations or vehicle for 1 h, followed by SNP incubation at 300 μM for 3 or 4 h. Preliminary studies were carried out to help determine the doses and the time points of the indicated treatments.

### Real-time assessment of cell death

To assess cell death, the cell-impermeant dsDNA-binding YOYO-1 iodide dye (Thermo Fisher Scientific, USA) was applied, followed by real-time quantification of YOYO-1-stained cells by Incucyte live cell analysis platform. Briefly, 4 h after the indicated treatments, 661W cells were incubated in 0.1 μM YOYO-1 iodide staining solution at 37 °C for 10 h. Four phase-contrast and fluorescent images per well were automatically captured at 1-h intervals. The integrated object counting algorithm was used to isolate the fluorescent nuclear signal from background. Specifically, images were segmented in order to identify individual objects, counted, and reported on a per-area basis for each time point. The confluence of green fluorescent signals was then measured by the Incucyte Live-Cell Analysis System (Sartorius, German).

### Assessment of mitochondrial membrane potential (MMP)

MMP was detected using the Mitochondrial Membrane Potential Assay kit (Beyotime, China). Briefly, 3 h after the indicated treatments, 661W cells were incubated in 5,5′,6,6′-tetrachloro-1,1′,3,3′-tetraethylbenzimidazolylcarbocyanine iodide (JC-1) detection solution at 37 °C for 20 min. The mitochondrial probe JC-1 is a lipophilic cationic dye that exhibits green fluorescence in the monomer forms. JC-1 dye can accumulate in the intact mitochondria and form the red fluorescent complex called J-aggregates that serve as a monitor of MMP [13]. The red fluorescence of J-aggregates and the green fluorescence of JC-1 monomers were acquired using a fluorescence microscope (DMI6000, Leica, Germany). Quantification of immunofluorescence was performed by ImageJ.

### Measurement of mitochondrial permeability transition pore (MPTP) opening

The opening of mPTP was detected using the Mitochondrial Permeability Transition Pore Assay kit (Beyotime, China). In brief, 3 h after the indicated treatments, cell-permeable calcein acetoxymethyl ester and CoCl_2_, the quencher of calcein fluorescence, were applied at 37 °C for 30 min to selectively label mitochondria. The green fluorescent signal of calcein was then visualized using a fluorescence microscope (DMI6000, Leica, Germany) and quantified by ImageJ.

### Measurement of mitochondrial superoxide production

The mitochondrial superoxide production was detected using the MitoSOX Red mitochondrial superoxide indicator (Thermo Fisher Scientific, USA). In brief, 3 h after the indicated treatments, 661W cells were incubated in the solution containing MitoSOX Red mitochondrial superoxide indicator at 37 °C for 30 min. The red fluorescent signal indicative of mitochondrial superoxide was acquired using a fluorescence microscope (DMI6000, Leica, Germany) and quantification was performed by ImageJ.

### Animals and treatments

Six-week-old female Balb/c mice were obtained from Shanghai Laboratory Animal Research Center and maintained in a laboratory with a 12/12 h light–dark cycle and a controlled temperature set at 20 ± 2 °C. The mice were dark-adapted for 24 h before the experimental light exposure (Compact Fluorescence Lamp, 45 W, Chaoya Lighting, Shanghai, China) delivered at 15,000 lx for 30 min. Hyperoside was dissolved in 0.5% sodium carboxymethyl cellulose solution and administered intraperitoneally 30 min before the light exposure at the indicated dose(s). Balb/c mice unexposed to the experimental light (normal controls) and the light-exposed mice without hyperoside treatment were treated with the vehicle in the same fashion. The volume of the intraperitoneal injection was controlled at 100 μL per mouse. Twenty-four mice from each experimental group were analyzed for the indicated imaging, electrophysiological, histopathological and molecular biological assessments at the specified time point(s). In total, 112 mice were utilized in the current study. The laboratory animal handling protocol was reviewed and approved by the Institutional Animal Care and Use Committee of Yueyang Hospital of Integrated Traditional Chinese and Western Medicine, Shanghai University of Traditional Chinese Medicine (YYLAC-2020-079-2 and YYLAC-2023-199-1) and carried out in accordance with the recommendations of the NIH Guide for the Care and Use of Laboratory Animals and the ARVO Statement for the Use of Animals in Ophthalmic and Vision Research.

### Optical coherence tomography (OCT)

Image-guided OCT (OCT 2 with Micron IV, Phoenix Research labs, USA) was adopted to image the retina 7 d after the experimental light exposure. In brief, anesthesia was induced by intraperitoneal injection of ketamine hydrochloride (82.5 mg/kg bw) and xylazine (8.25 mg/kg bw), followed by dilation of pupils using 1% tropicamide (Santen Pharmaceutical, Japan) prior to OCT imaging. Five full-retinal scans were acquired and automatically averaged using Phoenix Reveal OCT software (Phoenix Research labs, USA). The averaged scans were presented and subject to morphological evaluation of the retina. The thickness of the outer nuclear layer (ONL) was measured with Insight Image Segmentation Software for the Phoenix OCT and Retinal Imaging System (Version 2.0.5490, Voxeleron LLC, USA).

### Electroretinography (ERG)

Seven days after the experimental light exposure, the mice were dark-adapted for 24 h and subjected to ERG analysis under the safe light (5 lx) as previously described [14]. Briefly, prior to ERG procedures, intraperitoneal injection of ketamine hydrochloride (82.5 mg/kg bw) and xylazine (8.25 mg/kg bw) was performed to induce anesthesia. Pupils were then dilated using 1% tropicamide and the eyes were kept moisturized using 0.5% hypromellose solution. Once the mice were sedated, the reference and ground electrodes were inserted subcutaneously in the head in the midline between the ears and in the tail toward the base of the tail, respectively. The ERG responses were then recorded and analyzed by LabScribe software using Ganzfeld (ERG 2, Phoenix Research Labs, USA). Flashes of green light (504 nm) were delivered at the intensity of -2 (0.5 ms duration and 5 s inter-stimulus-interval), − 0.8 (1 ms duration and 5 s inter-stimulus-interval), 0.4 (1 ms duration and 10 s inter-stimulus-interval), 1.6 (1 ms duration and 20 s inter-stimulus-interval) and 3.1 (1 ms duration and 60 s inter-stimulus-interval) log cd·s·m^−2^.

### Histological examination and immunohistochemistry (IHC)

The enucleated eyes were fixed in 4% paraformaldehyde for 24 h before further processing and paraffin sectioning. Paraffin sections (4 μm in thickness) were stained with hematoxylin and eosin (HE) or subjected to IHC examination using primary antibodies including mouse anti-rhodopsin (1:1000) (Novus, USA), rabbit anti-opsin (1:100) (Red/Green, M-opsin) (Millipore, USA), rabbit anti-opsin (1:100) (Blue, S-opsin) (Millipore, USA) and rabbit anti-glial fibrillary acidic protein (GFAP) (1:500) (DAKO, USA) as well as Cy3-conjugated sheep anti-rabbit (1:1000) or sheep anti-mouse secondary antibodies (1:1000) (Sigma-Aldrich, USA). In addition, eye cups free of the cornea and lens were made and fixed in 4% paraformaldehyde for 2 h at room temperature and processed for cryosectioning. Cryosections (12 µm in thickness) were subjected to IHC examination using primary antibodies including rabbit anti-ionized calcium binding adaptor molecule 1 (Iba-1) (1:500) (Wako Chemicals, USA) and the secondary antibody Cy3-conjugated sheep anti-rabbit (1:1000). Counterstaining of 4-6-diamidino-2-phenylindole (DAPI) (Sigma Aldrich, USA) was performed for nuclei visualization. Microscopic imaging was performed by a light microscope (DM2000, Leica, Germany) or a fluorescent microscope (DM6000B, Leica, Germany). The value of gain and exposure time were maintained the same for the microscopic imaging of the fluorescent signals.

### Transmission electron microscope (TEM)

The superior part of the eye cup was dissected and fixed in 2.5% glutaraldehyde at 4 °C overnight. After washing off glutaraldehyde, the specimens were post-fixed in 1% osmic acid, dehydrated in ascending concentrations of ethanol, stained with 3% uranyl acetate and embedded in Epon 812 embedding fluid. Ultra-thin sections (70 nm in thickness) were then made and stained with lead citrate. Images were recorded digitally by a H-7650 transmission electron microscope (HITACHI, Japan).

### Terminal deoxynucleotidyl transferase-mediated dUTP nick-end labeling (TUNEL)

Enucleated eyes were fixed in 4% paraformaldehyde and processed for paraffin sectioning. Paraffin sections (4 μm in thickness) were then subjected to TUNEL assay (DeadEnd™ Fluorometric TUNEL System, Promega) following the manufacturer’s protocols. TUNEL positivity was observed and recorded using a fluorescent microscope (DM6000B, Leica, Germany). The value of gain and exposure time were maintained the same during the microscopic imaging process. ImageJ was used to quantify the TUNEL positivity.

### RNA sequencing

Total RNA was extracted from dissected retinas using mirVana miRNA Isolation Kit (Thermo Fisher Scientific, USA). RNA purity and quantification were evaluated by a NanoDrop 2000 spectrophotometer (Thermo Scientific, USA). Agilent 2100 Bioanalyzer (Agilent Technologies, Santa Clara, CA, USA) was used to assess RNA integrity. TruSeq Stranded mRNA LT Sample Prep Kit (Illumina, San Diego, CA, USA) was adopted to construct the cDNA libraries. The libraries were sequenced on an Illumina HiSeq X Ten platform and 150 bp paired-end reads were generated. Raw data (raw reads) of fastq format were processed using Trimmomatic [15] and the reads of low quality were removed to obtain the clean reads. Fragments per kilobase of transcript per million mapped reads (FPKM) of each gene was calculated using Cufflinks [16]. The read counts of each gene were obtained by HTSeq count [17]. Principal component analysis (PCA) was performed to evaluate the distribution and variation of the samples. The correlation heatmap was produced in R using the stats package. Differential expression analysis was performed using the DESeq (2012) R package. P value < 0.05 and fold change > 2 or fold change < 0.5 were set as the threshold for significantly differential expression. Hierarchical cluster analysis of differentially expressed genes (DEGs) was performed to demonstrate the expression pattern of genes in the indicated experimental groups and individual samples. Gene set enrichment analysis (GSEA) was performed for functional enrichment of the genes using Kyoto Encyclopedia of Genes and Genomes (KEGG) and Gene Ontology (GO) gene sets with ClusterProfiler R package and org.Mm.eg.db annotation package. The gene sets with significant enrichment were defined with absolute value of normalized enrichment score (NES) > 1 and false discovery rate (FDR) q-value < 0.05. Benjamini–Hochberg procedure was used for the correction of the related FDR q-value. The bubble plot of GSEA core-enriched signaling pathways was generated with ggplot2 in R package.

### Real-time qPCR analysis

TRIzol reagent (Invitrogen, USA) was used for isolating RNA from the mouse retinas. PrimeScript RT Master Mix (TaKaRa, Japan) was then used for reverse transcription. The expression of *Abca4*, *Aim2*, *Axl*, *Bbs9*, *Casp4*, *Casp8*, *Ccl2*, *Cd68*, *Clec7a*, *Cnga1*, *Crx*, *Gfap*, *Glul*, *Gnat1*, *Guca1a*, *Il1b*, *Mlkl*, *N2re3*, *Naip2*, *Nlrp3*, *Nrl*, *Nxnl1*, *Opn1mw*, *Opn1sw*, *P2ry12*, *Pde6b*, *Prph2*, *Rdh12*, *Reep6*, *Rho*, *Ripk1*, *Ripk3*, *Rom1*, *Rp1l1*, *Rpgrip1*, *Slc24a1*, *Tmem119*, *Tnf*, *Tspo* and *Ush2a* was examined using SYBR Green I Master (Roche, USA) on a Roche Light Cycler 480 II (Roche, USA). The primer sequences were included in Table 1. The expression of the indicated genes was normalized to *18S rRNA*. The fold change of gene expression was calculated based on 2^−[Ct (candidate gene)−Ct (18s rRNA)]^.

**Table 1:** Primer sequences for real-time qPCR analysis

Gene name	Forward primer (5′–3′)	Reverse primer (5′–3′)
*Abca4*	CAGAAGATTCGCTTTGTAGTGGA	CCTTGTTGGGAAAATGGCATTC
*Aim2*	GTCACCAGTTCCTCAGTTGTG	CACCTCCATTGTCCCTGTTTTAT
*Axl*	ATGGCCGACATTGCCAGTG	CGGTAGTAATCCCCGTTGTAGA
*Bbs9*	AGCCACCAATGTGGAACCTGGA	CTGTAGTGGAGGCTGCACATAG
*Casp4*	GTGGTGAAAGAGGAGCTTACAGC	GCACCAGGAATGTGCTGTCTGA
*Casp8*	CGGTGAAGAACTGCGTTTCC	ACGCCAGTCAGGATGCTAAG
*Ccl2*	AGCTGTAGTTTTTGTCACCAAGC	GTGCTGAAGACCTTAGGGCA
*Cd68*	GGCGGTGGAATACAATGTGTCC	AGCAGGTCAAGGTGAACAGCTG
*Clec7a*	GACTTCAGCACTCAAGACATCC	TTGTGTCGCCAAAATGCTAGG
*Cnga1*	CGAGCCATTTGTGCTGCTTA	TCATGGTTAGTTTAATATCTGCGCT
*Crx*	CCAATGTGGACCTGATGCACCA	GTACTGGGTCTTGGCAAACAGG
*Gfap*	CCGAGTACTGAAGCCAAGGG	GCAGTTTGTAACCCCTCCCA
*Glul*	GAGGAGAATGGTCTGAAGTGC	ACCGGCAGAAAAGTCGTTGA
*Gnat1*	CCCGACTACGATGGACCTAAC	TTGACGTTCTGTGTGTCGGT
*Guca1b*	CTGGACATTGTGGAGGCGAT	GACAGCTGGCCGTCTCCATT
*Il1b*	TGCCACCTTTTGACAGTGATG	AAGGTCCACGGGAAAGACAC
*Mlkl*	CTGAGGGAACTGCTGGATAGAG	CGAGGAAACTGGAGCTGCTGAT
*N2re3*	GCCTTATCACCGCCGAAACTTG	CATGGATGCCATCCAGACTGCA
*Naip2*	AGCTTGGTGTCTGTTCTCTGT	GCGGAAAGTAGCTTTGGTGTAG
*Nlrp3*	ATTACCCGCCCGAGAAAGG	TCGCAGCAAAGATCCACACAG
*Nrl*	CTCTTGGCTACTATTCAGGGAGC	GGTTCAACTCGCGCACAGACAT
*Nxnl1*	GGAACAACAGCGACCAGGAT	GTGAGCCGCACGAAGAAGT
*Opn1mw*	AGCCCTTTGGCAATGTGAGA	AAGGCCAGTACCTGCTCCAA
*Opn1sw*	TCATCTTCTGTTTCATCATTCCTCT	CTTTTGTGTCGTAGCAGACTCTT
*P2ry12*	ATGGATATGCCTGGTGTCAACA	AGCAATGGGAAGAGAACCTGG
*Pde6b*	TGGAGAACCGTAAGGACATCGC	TCCTCACAGTCAGCAGGCTCTT
*Prph2*	GCAATCGCTACCTGGACTTCTC	GTGAGCTGGTACTGGATACAGG
*Rdh12*	ATCTTGGTACTGCTTACGTCCT	CACCAGCAAAGAACTTCCTGA
*Reep6*	CGGTTACGGGGCCTCTCTA	CCAGTAGGTTAGCCACACAGT
*Rho*	CCTTTGTCATCTACATGTTCGTGGT	CTTCCTTCTCTGCCTTCTGAGTGGT
*Ripk1*	GACTGTGTACCCTTACCTCCGA	CACTGCGATCATTCTCGTCCTG
*Ripk3*	CCACACCAGCAAGGACATCT	GCCGAACTTGAGGCAGTAGT
*Rom1*	TGGGTCAGCAACCGTTACTTGG	GAGAGTTGGCTTTGCAGGCAAG
*Rp1l1*	TGTGACTGCGAGGAGTGAACGT	TCAAGGAAACCTGCCGCAGCTT
*Rpgrip1*	GACCACGAAGAGAAACTGGAGC	CAGAGTGCCATACGCGACATCT
*Slc24a1*	GTCAAGGTCTGAAGGTTTGGG	TCTTTGGTCGGAGTAACCGC
*Tmem119*	CCTACTCTGTGTCACTCCCG	CACGTACTGCCGGAAGAAATC
*Tnf*	ACGTCGTAGCAAACCACCAA	GCAGCCTTGTCCCTTGAAGA
*Tspo*	GAGCCTACTTTGTACGTGGCGA	GCTCTTTCCAGACTATGTAGGAG
*Ush2a*	TGCTCAGTGACCCTGTTTCCGT	TTGTTGCGAGCTGGTGTAGACC
*18S rRNA*	GAGGTTCGAAGACGATCAGA	TCGCTCCACCAACTAAGAAC

### Statistical analysis

Data were expressed as mean ± standard error of mean (SEM) or mean ± standard deviation (SD). Statistical analyses were performed by one-way or two-way ANOVA with the Tukey’s multiple comparisons test (GraphPad Prism 9, USA). Statistically significance was defined if *P* < 0.05.

## Results

### Hyperoside alleviates oxidative stress-induced photoreceptor cell death in part by mitigating mitochondrial dysfunction

**Fig. 1:**
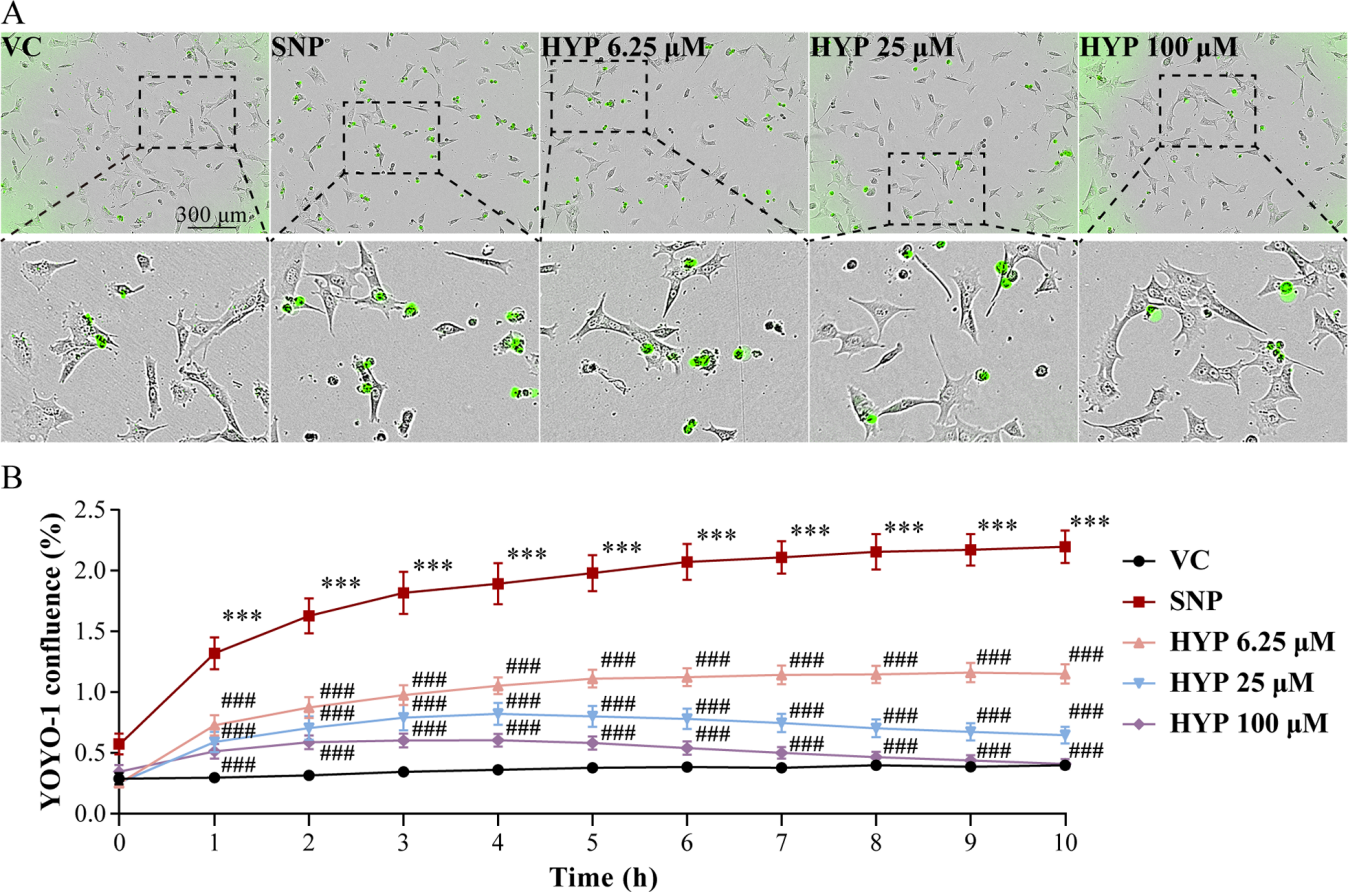
Hyperoside inhibits SNP-induced photoreceptor cell death. 661W cells pretreated with vehicle or hyperoside at the indicated concentrations were incubated in the presence or absence of 300 μM SNP for 4 h. **A** YOYO-1 positivity (in green) recorded by Incucyte live cell analysis platform 10 h after the termination of SNP stimulation. Scale bar, 300 μm. **B** Quantification of YOYO-1 confluence at 1-h intervals using Incucyte Live-Cell Analysis System. Data were expressed as mean ± SEM. (n = 6 per group). ***Compared to VC, *P* < 0.001; ^###^compared to SNP, *P* < 0.001. VC, the vehicle-treated cells without SNP exposure; SNP, the SNP-stimulated vehicle-treated cells; HYP, the SNP-stimulated cells treated with hyperoside

**Fig. 2:**
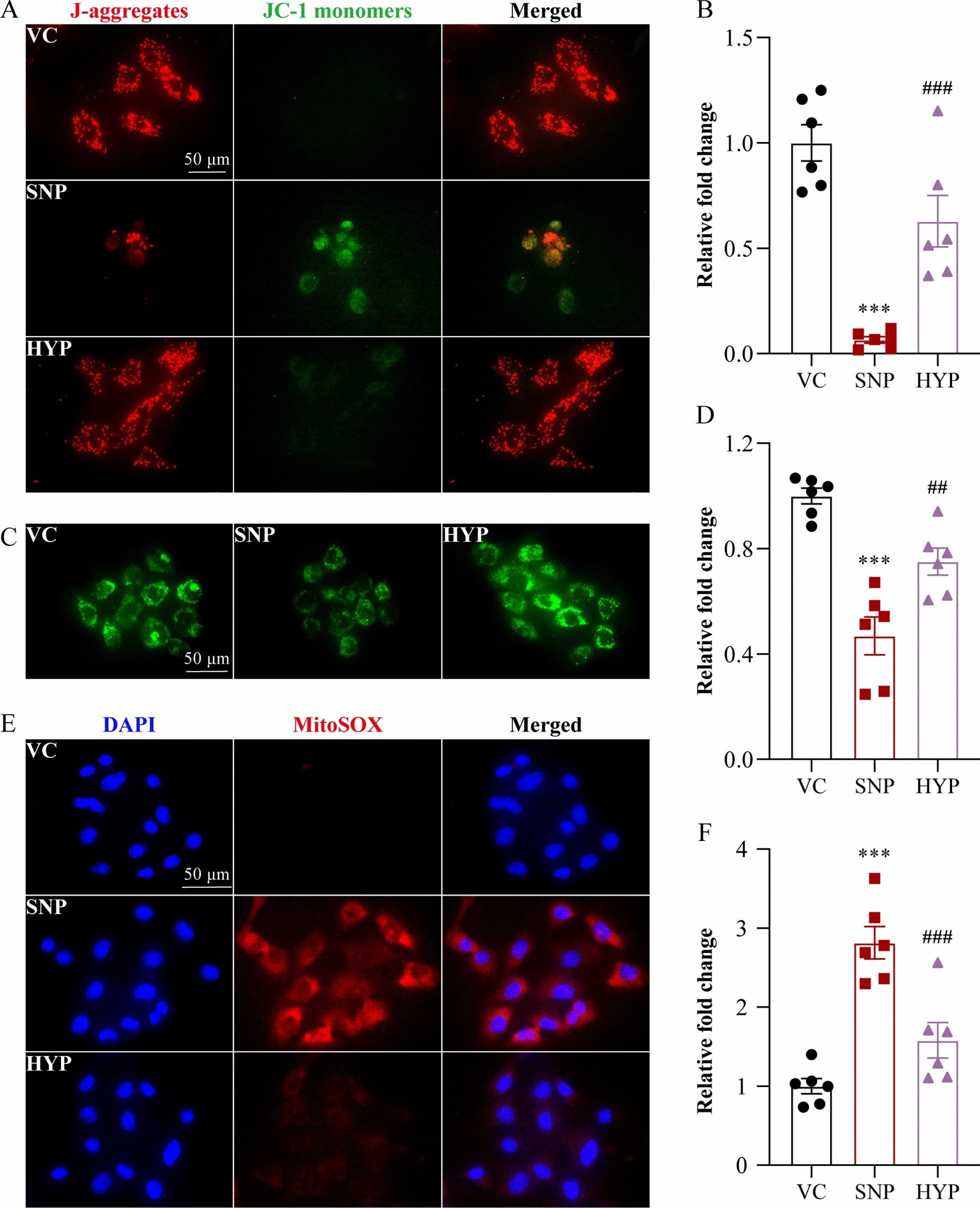
Hyperoside protects against SNP-induced mitochondrial dysfunction in photoreceptor cells. 661W cells pretreated with vehicle or 100 μM hyperoside were incubated in the presence or absence of 300 μM SNP for 3 h. **A** The mitochondria probe JC-1 dye was applied, followed by microscopic imaging of J-aggregates (in red) and JC-1 monomers (in green) by fluorescence microscopy. DAPI (in blue) was counterstained to visualize the nuclei. Scale bar, 50 μm. **B** The relative fold change in the ratio of J-aggregates to JC-1 monomers was plotted against VC (n = 6 per group). **C** Representative microscopic image showing calcein positive fluorescent signals. Scale bar, 50 μm. **D** Relative fold change in the calcein positive fluorescence intensity was plotted against VC (n = 6 per group). **E** Representative microscopic images showing MitoSOX Red positive fluorescent signals. Scale bar, 50 μm. **F** Relative fold change in the MitoSOX Red positive fluorescence intensity was plotted against VC (n = 6 per group). Data were expressed as mean ± SEM. ***Compared to VC, *P* < 0.001; ^##^compared to SNP, *P* < 0.01; ^###^compared to SNP, *P* < 0.001. VC, the vehicle-treated cells without SNP exposure; SNP, the SNP-stimulated cells treated with vehicle; HYP, the SNP-stimulated cells treated with hyperoside

To directly assess the hypothesis that hyperoside protects against oxidative stress-induced mitochondrial impairment and cell death, 661W cells were subjected to SNP treatment, which has been shown to induce photoreceptor cell death by triggering reactive oxygen species (ROS) production in mitochondria [12]. Real-time live-cell analysis revealed that SNP induced significant increases in YOYO-1 positivity in the vehicle-treated cells over time. In sharp contrast, much lower YOYO-1 positivity was observed during the course of 10-h SNP stimulation when hyperoside was applied at 6.25, 25 and 100 μM (Fig. 1A, B). These results support the cell-intrinsic effect of hyperoside against oxidative stress-induced photoreceptor cell death. Next, mitochondrial function and mitochondrial superoxide production were examined to further address the hypothesis that hyperoside may protect against oxidative stress-induced photoreceptor cell death by mitigating mitochondrial impairment. Significantly decreased ratio of J-aggregates to JC-1 monomers was observed in the vehicle-treated SNP-stimulated cells. Hyperoside treatment, however, resulted in increased ratio of J-aggregates to JC-1 monomers in the SNP-stimulated cells (Fig. 2A, B). Under normal circumstance, the cationic JC-1 dye enters and accumulates in the negatively charged mitochondria, resulting in the formation of the red fluorescent J-aggregates. In contrast, in the unhealthy or dying cells, the mitochondria are less negatively charged due to the loss of MMP. As a result, the ability of the JC-1 dye to enter the mitochondria is impaired, leading to less J-aggregates formation and increased presence of green fluorescent JC-1 monomers [13]. Therefore, increased ratio of J-aggregates to JC-1 monomers in the hyperoside-treated SNP-stimulated cells indicate that hyperoside protects against SNP-induced loss of MMP in the photoreceptors. During cell death, loss of MMP results from continuous opening of the MPTP. Therefore, MPTP was subsequently assessed. SNP stimulation led to the opening of MPTP as shown by reductions in the calcein positive signals in the mitochondria. On the contrary, increased calcein positivity was observed in the SNP-stimulated cells treated with hyperoside (Fig. 2C, D). Furthermore, the opening of MPTP results from increased levels of ROS in mitochondria, an unavoidable consequence of mitochondrial dysfunction. Thus, mitochondrial superoxide formation was also examined. As shown in Fig. 2E, F, SNP stimulation increased the level of superoxide in the mitochondria. In contrast, lower level of mitochondrial superoxide was observed in the hyperoside-treated SNP-stimulated cells. Taken together, these results demonstrate that the cell-intrinsic photoreceptor protective effects of hyperoside in part implicate mitigating oxidative stress-induced mitochondrial dysfunction.

### Hyperoside protects against photooxidative stress-mediated photoreceptor morphological degeneration and retinal functional decline

**Fig. 3:**
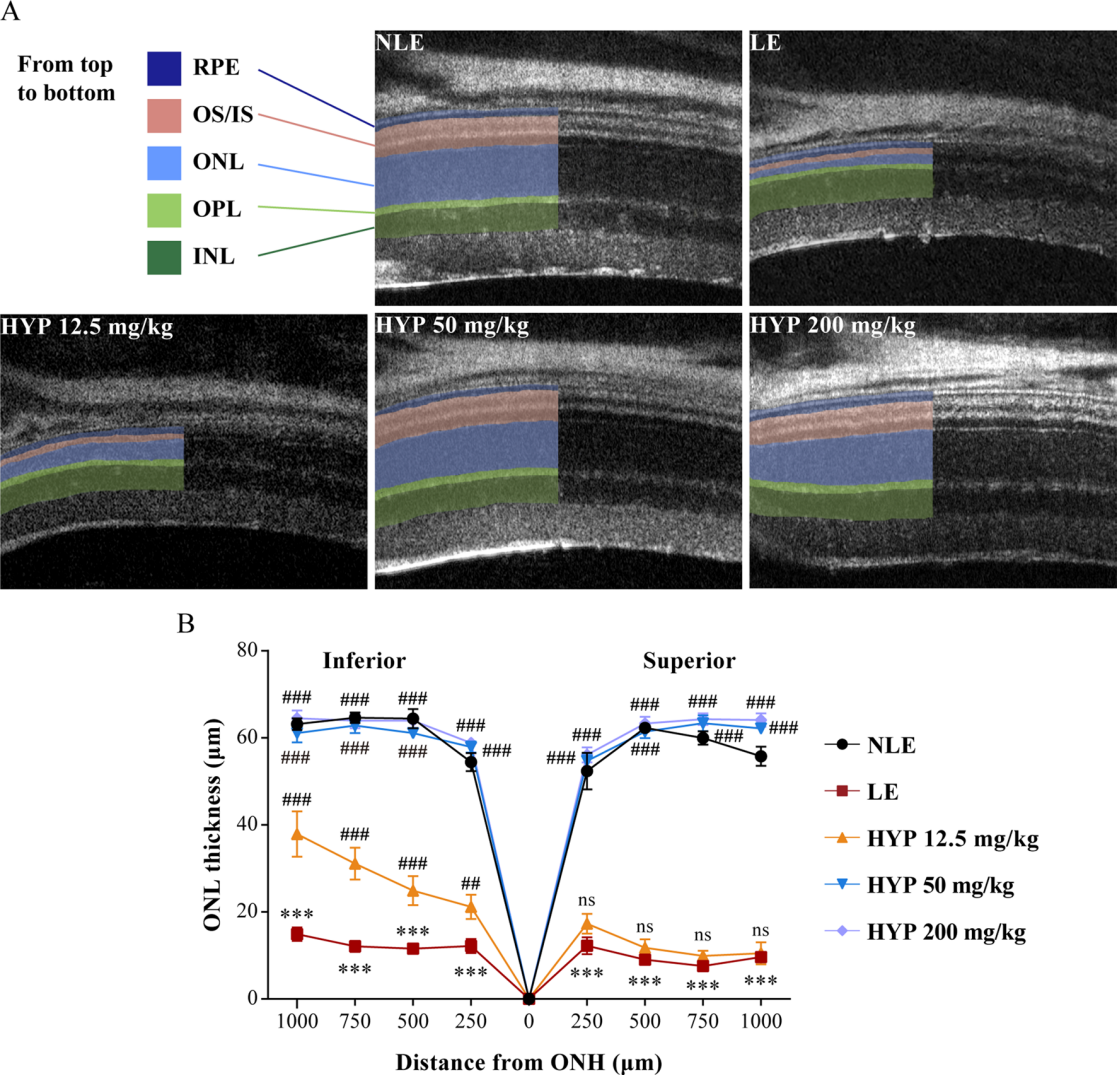
Hyperoside protects against light-induced photoreceptor morphological degeneration. **A** Representative OCT scans from the superior retinas. **B** The ONL thickness measured at 250, 500, 750 and 1000 μm from ONH in the inferior and superior retinas. Data were expressed as mean ± SEM (n = 6 per group). ***Compared to NLE, *P* < 0.001; ^##^compared to LE, *P* < 0.01, ^###^compared to LE, *P* < 0.001; ns, not significant. *HYP-L* the light-exposed mice treated with 12.5 mg/kg hyperoside, *HYP-M* the light-exposed mice treated with 50 mg/kg hyperoside, *HYP-H* the light-exposed mice treated with 200 mg/kg hyperoside, *INL* inner nuclear layer, *IS* inner segment, *LE* the light-exposed mice treated with vehicle, *NLE* the vehicle-treated mice without light exposure, *ONH* optic nerve head, *ONL* outer nuclear layer, *OPL* outer plexiform layer, *OS* outer segment, *RPE* retinal pigment epithelium

**Fig. 4:**
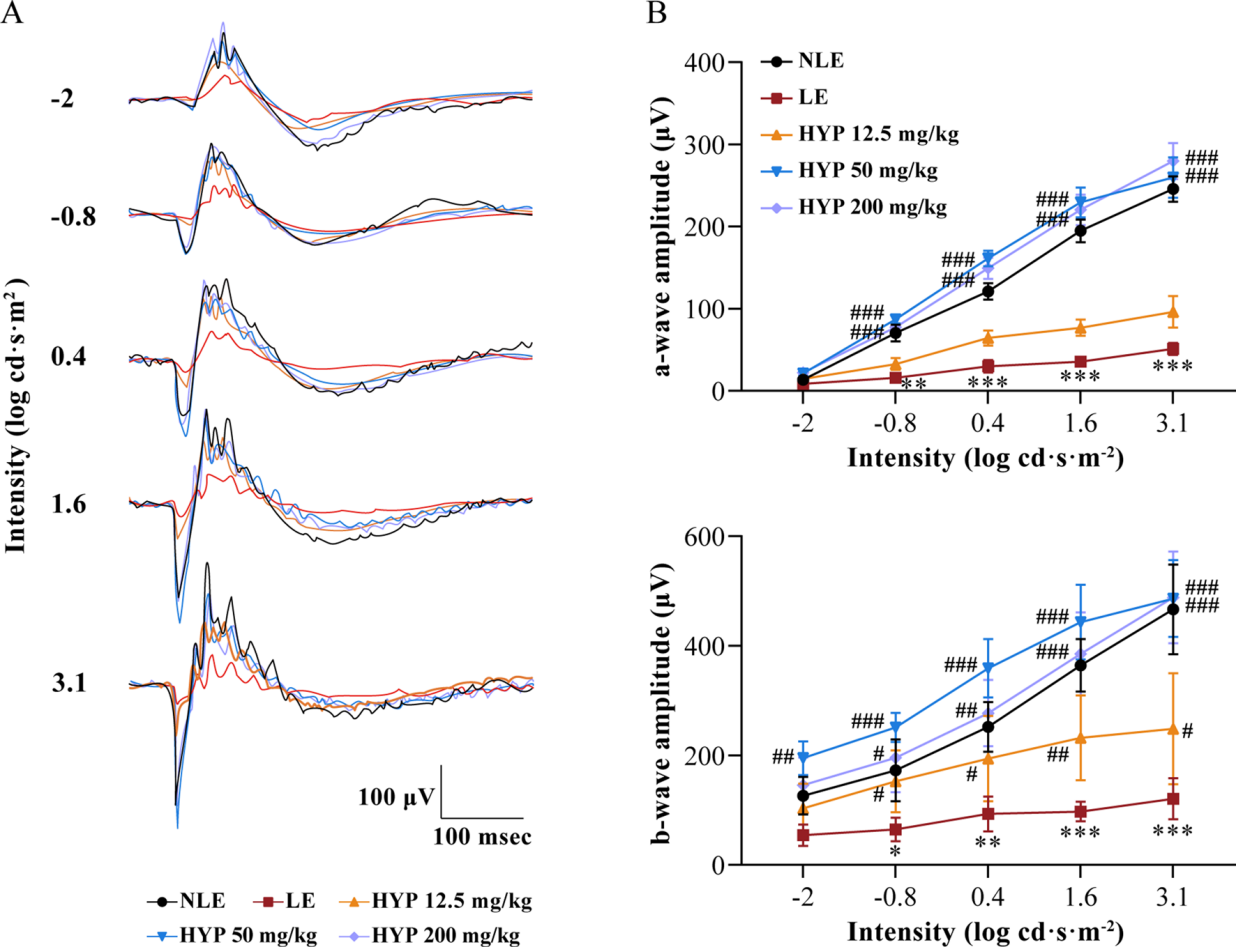
Hyperoside protects against light-induced decline of the retinal function. **A** Representative scotopic electroretinograms. **B** Amplitudes of a-wave and b-wave were plotted. Data were expressed as mean ± SD (n = 6 per group). *Compared to NLE, P < 0.05; **compared to NLE, P < 0.01; ^#^compared to LE, P < 0.05; ^##^compared to LE, P < 0.01. *HYP-L* the light-exposed mice treated with 12.5 mg/kg hyperoside, *HYP-M* the light-exposed mice treated with 50 mg/kg hyperoside, *HYP-H* the light-exposed mice treated with 200 mg/kg hyperoside, *LE* the light-exposed mice treated with vehicle, *NLE* the vehicle-treated mice without light exposure

Next, to directly verify the protective effects of hyperoside against oxidative stress-mediated photoreceptor degeneration in vivo, photooxidative stress-mediated photoreceptor degeneration was modeled in Balb/c mice. Seven days after the experimental light exposure, full-retinal morphological views were obtained from OCT imaging to gain an objective assessment of the pharmacological effects of hyperoside on the photoreceptor morphology (Fig. 3A). The thickness of the photoreceptor outer nuclear layer (ONL) was then measured to provide a quantitative understanding of the changes associated with the photoreceptor morphology (Fig. 3B). In sharp contrast to the intact retinal structures documented from the vehicle-treated mice without light exposure (NLE), the retinas from the vehicle-treated light-exposed (LE) mice were characterized by a marked diminishment of the ONL, inner segment (IS) and outer segment (OS). However, well-maintained photoreceptor morphology was observed in the light-exposed mice treated with 50 and 200 mg/kg hyperoside. In the meantime, the photoreceptor morphology was partially preserved in the inferior retina in the light-exposed mice treated with 12.5 mg/kg hyperoside (Fig. 3A, B). Similar results were obtained when the retinal function was examined by ERG recording. As shown in Fig. 4A, B, scotopic ERG analyses revealed that the a-wave and b-wave, the negative and positive waveforms reflecting the functionality of photoreceptors and the second-order retinal neurons, respectively, exhibited remarkable depression in their amplitudes in the LE mice compared to the NLE mice. In contrast, the a-wave and b-wave amplitudes were significantly increased in the light-exposed mice treated with 50 and 200 mg/kg hyperoside compared to the LE mice. In keeping with the partial protection of the photoreceptor morphology revealed by OCT imaging (Fig. 3), the a-wave and b-wave responses were partially preserved in the light-exposed mice treated with 12.5 mg/kg hyperoside. Taken together, the results from OCT and ERG analyses provide direct in vivo evidence supporting that hyperoside protects against photooxidative stress-mediated photoreceptor morphological degeneration and associated functional impairment of the retina.

### Hyperoside preserves the morphological integrity of photoreceptors under photooxidative stress conditions

**Fig. 5:**
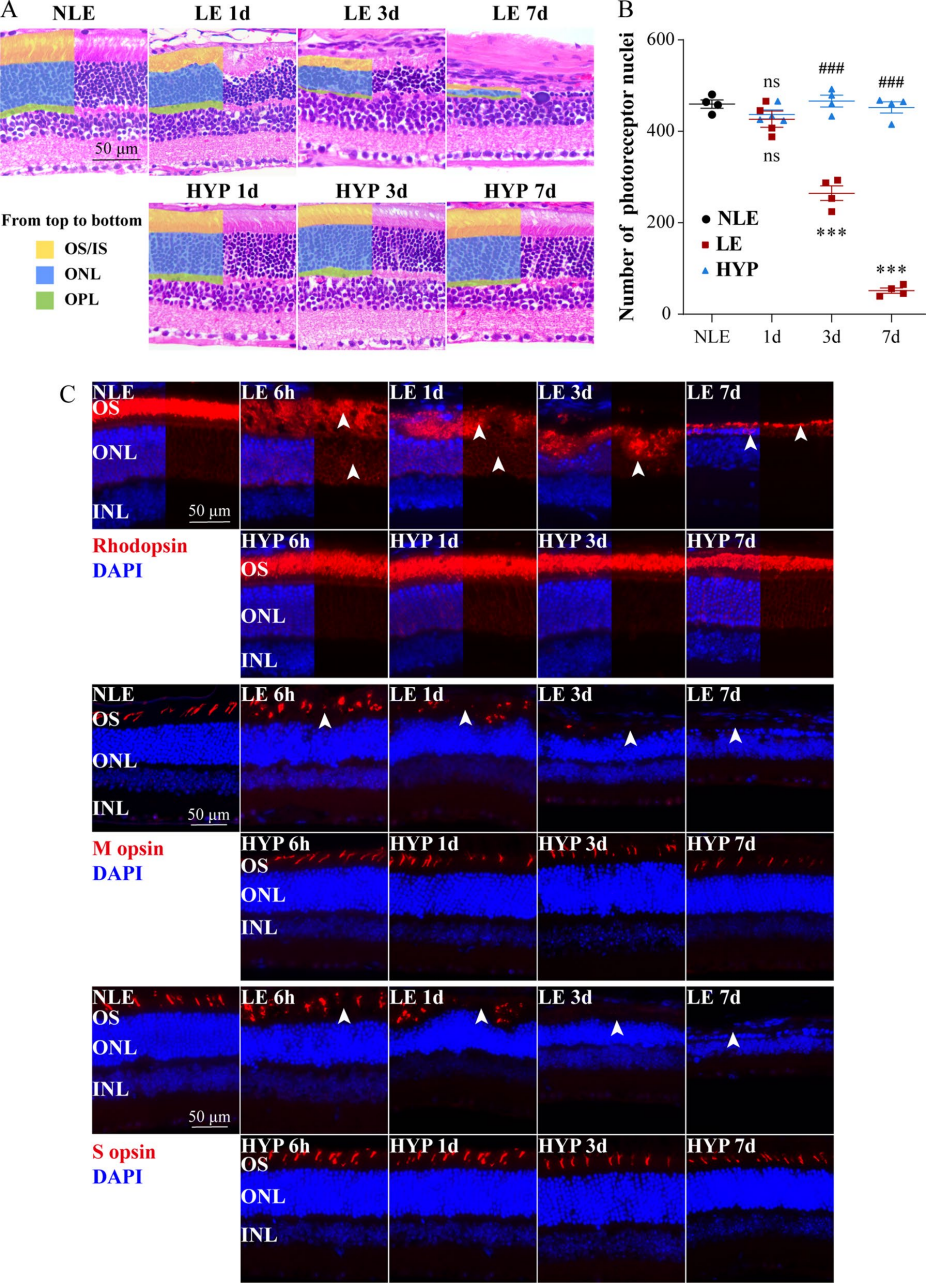
Hyperoside protects against light-induced impairment of photoreceptor morphological integrity. **A** Representative HE-stained microscopic images. Scale bar, 50 μm. **B** The number of photoreceptor nuclei was counted at 500 μm away from ONH in the superior retina. **C** Representative microscopic images showing the expression pattern of rhodopsin, M-opsin and S-opsin (in red) in the retina. DAPI (in blue) was counterstained to label the nuclei. White arrowheads indicated disordered, mislocalized or residual rhodopsin expression as well as mislocalized or absence of M-opsin and S-opsin expression. Scale bar, 50 μm. Data were expressed as mean ± SEM (n = 4 per group). ***Compared to NLE, *P* < 0.001; ^###^compared to LE, *P* < 0.001; ns, not significant. *HYP* the light-exposed mice treated with 50 mg/kg hyperoside, *INL* inner nuclear layer, *IS* inner segment, *LE* the light-exposed mice treated with vehicle, *NLE* the vehicle-treated mice without light exposure, *ONL* outer nuclear layer, *OS* outer segment

Histological and immunohistochemical examination was subsequently performed to further characterize the protective effects of hyperoside on photoreceptors. Given that a nearly complete protection of the photoreceptor structure and retinal function was observed when hyperoside was administered at 50 mg/kg (Figs. 3 and 4), this dose was repeated for the following experiments. First, the eye cups collected during the course of photoreceptor degeneration, specifically, 1d, 3d and 7d post illumination, were subjected to histological examination. As shown in Fig. 5A, disarrangement of the photoreceptor IS and OS, disappearance of the demarcation between IS and OS and disorganization of the photoreceptor ONL were notable in the LE retinas 1d after the experimental light exposure. By 3d post illumination, remarkably shortened IS/OS and partial loss of photoreceptors were observed in the LE retinas. By 7d post illumination, the photoreceptor IS and OS were barely observed in the LE retinas and the number of photoreceptor nuclei in the ONL was significantly lower (Fig. 5A, B). In sharp contrast, the morphological features of the photoreceptor IS, OS and ONL in the light-exposed hyperoside-treated (HYP) retinas remained comparable to the NLE retinas at all the time points examined (Fig. 5A). Consistently, significantly higher number of photoreceptor nuclei was observed in the ONL in the HYP retinas compared to the LE retinas (Fig. 5B).

In addition, the in situ expression of rod photoreceptor-specific rhodopsin as well as cone photoreceptor-specific M-opsin and S-opsin was assessed to visualize the protective effects of hyperoside on rod and cone photoreceptors, respectively. For this purpose, cryosections were made 6 h, 1d, 3d and 7d after illumination and subjected to IHC examination. As shown in Fig. 5C, disorganized expression pattern of rhodopsin was noted in the LE retinas 6 h, 1d and 3d post illumination, failing to clearly outline the OS compartment of photoreceptors as observed in the NLE retinas. Meanwhile, rhodopsin immunopositivity was also readily detected in the ONL in the LE retinas collected 6 h and 1d after illumination, implying rhodopsin mistrafficking/mislocalization. By 7d post illumination, only residual expression of rhodopsin was detected in the LE retinas. In addition, disorganized expression pattern of M-opsin and S-opsin was observed in the LE retinas 6 h and 1d after illumination. By 3d after the experimental light exposure, the expression of M-opsin and S-opsin was barely detected in the LE retinas. By 7d post illumination, the expression of M-opsin and S-opsin was nearly undetectable in the LE retinas. In contrast, the expression pattern of rhodopsin, M-opsin and S-opsin in the HYP retinas remained similar to the pattern observed in the NLE retinas at all the time points examined.

**Fig. 6:**
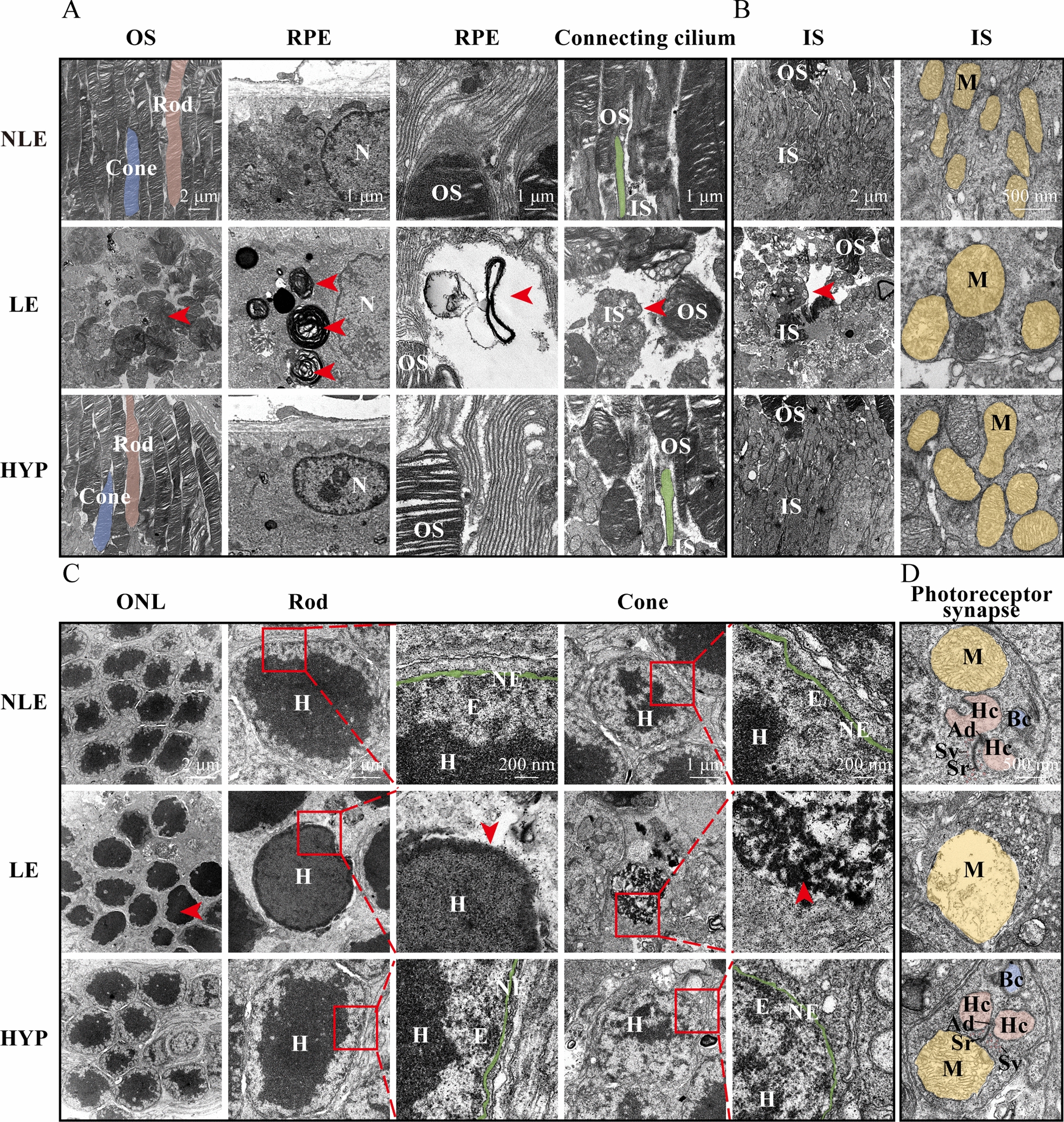
Hyperoside protects against light-induced photoreceptor ultrastructural impairment. **A** Representative TEM images of rod (pink) and cone (blue) photoreceptor OS, RPE and connecting cilium (green). Red arrowheads, disordered OS (first column); RPE phagosomes (second column); disrupted microvilli (third column); impaired connecting cilium (fourth column). Scale bars, 2 μm (first column); 1 μm (second, third and fourth column). **B** Representative TEM images of the IS and mitochondria in the IS (yellow). Red arrowhead, disrupted IS. Scale bars, 2 μm (first column) and 500 nm (second column). **C** Representative TEM images showing rod and cone photoreceptor nuclei. Red arrowheads, chromatin condensation (first column), damaged nuclear envelope (third column) and chromatin depolymerization (fifth column). Scale bars, 2 μm (first column), 1 μm (second and fourth column) and 200 nm (third column). **D** Representative TEM images highlighting photoreceptor synaptic terminals. Scale bar, 500 nm. *Ad* arciform density, *Bc* bipolar cell, *Hc* horizontal cell, *HYP* the light-exposed mice treated with 50 mg/kg hyperoside, *IS* inner segment, *LE* the light-exposed mice treated with vehicle, *M* mitochondria, *N* nucleus, *NLE* the vehicle-treated mice without light exposure, *OS* outer segment, *Sr* synapse ribbon, *Sv* synaptic vesicle

Next, to validate the protective effects of hyperoside on photoreceptors in greater detail, the retinal ultrastructure was examined by TEM. First, in distinct contrast to the normal ultrastructural features observed in photoreceptors from the NLE mice, overtly disorganized OS membrane discs and disrupted connecting cilium were readily observed in the LE retinas. Secondly, phagocytosed membrane discs were evident in the neighboring retinal pigment epithelium (RPE). The apical microvilli of RPE cells also became irregularly shaped and disorganized (Fig. 6A). Thirdly, the ultrastructure of the photoreceptor IS was also evidently damaged. In particular, the mitochondria in the IS were swollen and manifested disrupted cristae (Fig. 6B). Fourthly, in the photoreceptor nuclei, diminishment of the peripherally distributed euchromatin and loss of the integrity of the nuclear envelop were prominent (Fig. 6C). Lastly, in the photoreceptor synapses, the synaptic ribbons of photoreceptors and dendritic endings of the second-order horizontal and bipolar cells were readily detected; the typical triad structure was evident in the photoreceptors from the NLE mice. In contrast, the synaptic ribbon and dendritic endings were severely damaged and the typical triad structure was barely detected in the photoreceptor synapses from the LE mice. Prominent disruption of the cristae and vacuolation of mitochondria were also readily detected in the photoreceptor synapses in the LE mice (Fig. 6D). In sharp contrast to the above-mentioned ultrastructural abnormalities found in the LE photoreceptors and neighboring RPE cells, hyperoside treatment resulted in well-maintained ultrastructural features in photoreceptor structural compartments including the OS, IS, nuclei and synaptic terminals as well as in neighboring RPE cells (Fig. 6).

Everything considered, the results from the histological, immunohistochemical and ultrastructural examinations demonstrate that hyperoside confers significant protection to the photoreceptor morphological integrity under photooxidative stress conditions.

### Hyperoside protects against photooxidative stress-triggered whole-genome gene expression alterations in the retina

**Fig. 7:**
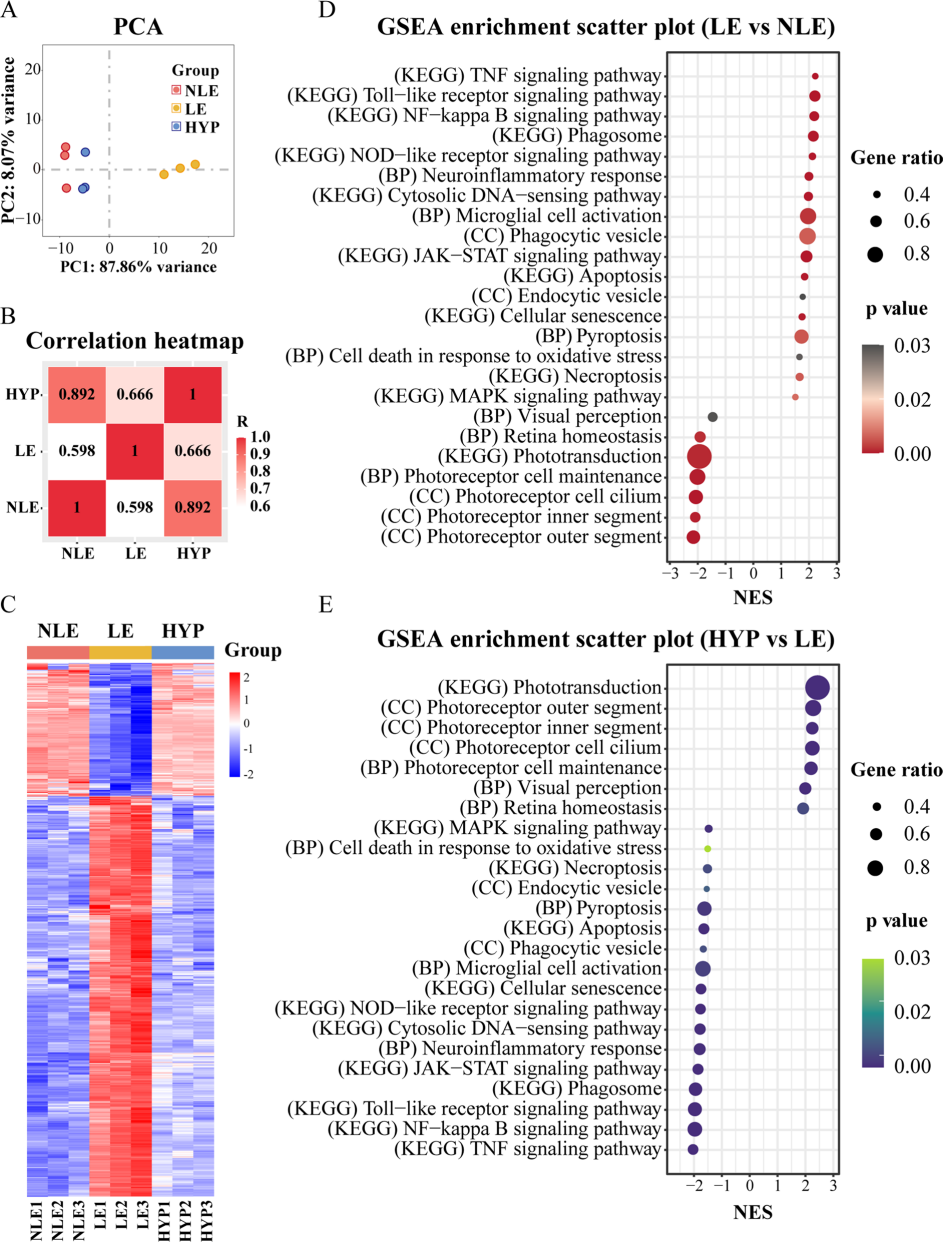
Hyperoside treatment counteracts light-induced alterations in the retinal gene expression. **A** PCA of RNA-seq data. **B** Correlation heatmap of DEGs. R, the Kendall correlation coefficient. **C** Heat map generated from hierarchical clustering analysis of the DEGs. **D** GSEA enrichment scatter plot (LE vs NLE). **E** GSEA enrichment scatter plot (HYP vs LE). *NES* normalized enrichment score, *HYP* the light-exposed mice treated with 50 mg/kg hyperoside, *LE* the light-exposed mice treated with vehicle, *NLE* the vehicle-treated mice without light exposure

To further understand the molecular mechanisms associated with the photoreceptor protective effects of hyperoside, the retinas were collected 1d post illumination, a time point prior to significant loss of photoreceptors were noted (Fig. 5A), and subjected to RNA-seq analyses to characterize the whole-genome gene expression profiles. The PCA of the RNA-seq data unveiled that the overall transcriptomic patterns were similar between the NLE retinas and the HYP retinas, which were explicitly different from the LE retinas (Fig. 7A). Pearson’s correlation analysis of the DEGs revealed a higher correlation coefficient between the NLE and the HYP retinas. Lower correlation coefficients were yielded when the comparison was made between the LE retinas and the NLE retinas or between the LE retinas and the HYP retinas (Fig. 7B). Hierarchical clustering of the DEGs demonstrated that compared to the NLE retinas, 730 genes were significantly upregulated and 300 genes were significantly downregulated in the LE retinas. Out of the 730 upregulated genes associated with the LE retinas, 626 genes were significantly downregulated in the HYP retinas. Meanwhile, out of the 300 downregulated genes found in the LE retinas, 198 genes were significantly upregulated in the HYP retinas (Fig. 7C). GSEA further revealed that compared to the NLE retinas, multiple molecular pathways associated with inflammatory responses (e.g. TNF signaling pathway, toll-like receptor signaling pathway, NF-kappa B signaling pathway, neuroinflammatory responses, cytosolic DNA-sensing pathway, microglial cell activation, etc.) and programmed cell death (e.g. apoptosis, necroptosis, pyroptosis and cell death in response to oxidative stress) were significantly upregulated in the LE retinas. In the meantime, the pathways closely linked to the photoreceptor morphology (e.g. axoneme, photoreceptor cell cilium, photoreceptor inner segment and photoreceptor outer segment), phototransduction, photoreceptor cell maintenance, visual perception, chromatin silence and retina homeostasis were significantly downregulated in the LE retinas (Fig. 7D). In contrast, hyperoside treatment significantly offset the deleterious impact of photooxidative stress on the above-mentioned pathways (Fig. 7E). Thus, the results from the RNA-seq analyses provide a non-biased overview of the molecular pathways associated with the protective effects of hyperoside against photooxidative stress-mediated photoreceptor degeneration.

### Hyperoside maintains the retinal expression of the genes essential for the morphological and functional integrity of photoreceptors under photooxidative stress conditions

**Fig. 8:**
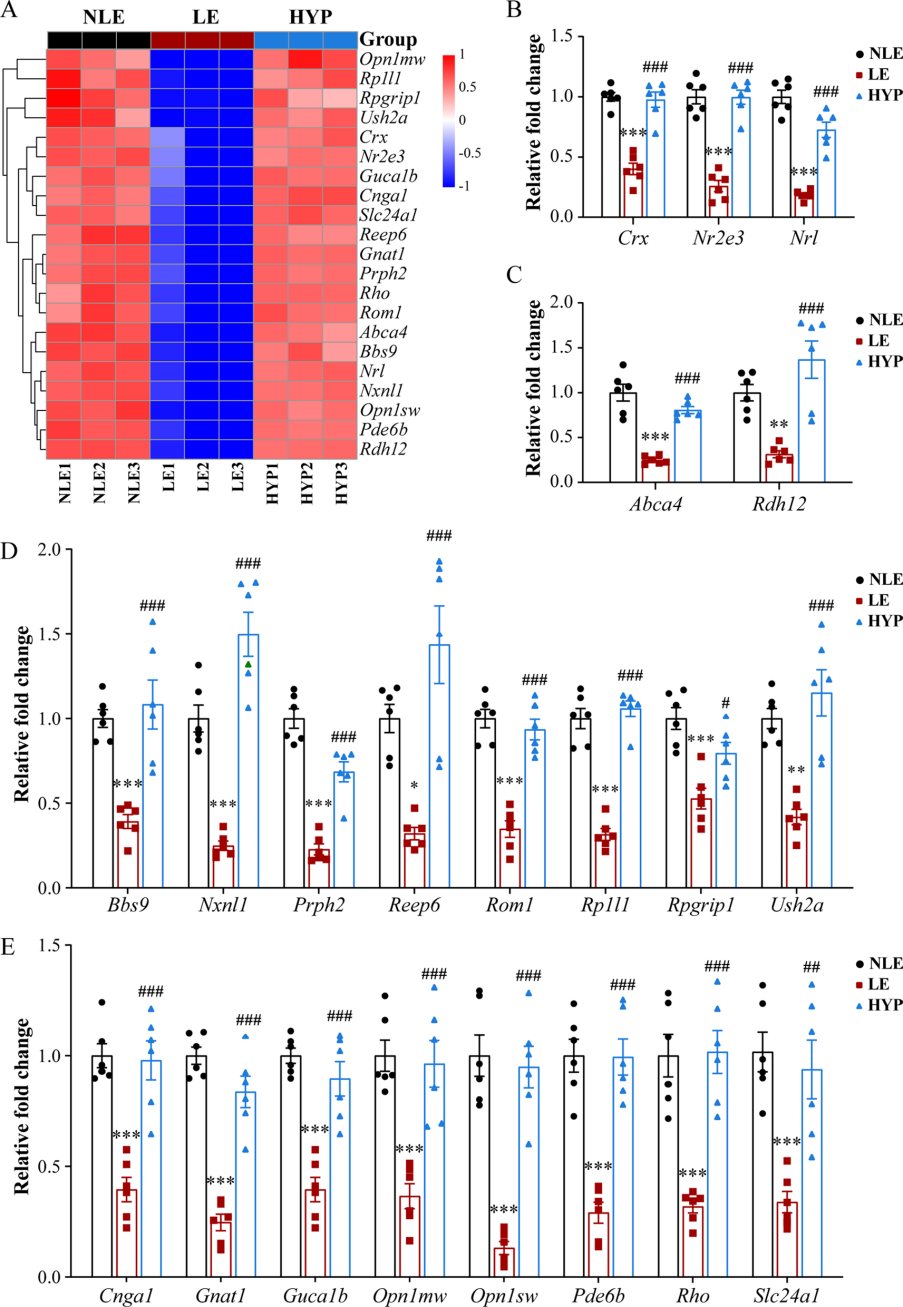
Hyperoside attenuates light-induced dysregulation in the retinal expression of genes essential for photoreceptor morphological and functional integrity. **A** Heatmap visualization of the representative genes important for photoreceptor morphological and functional integrity revealed by RNA-seq analyses. **B**–**E** Real-time qPCR validation of the expression of transcription factors essential for photoreceptor identity (**B**), the genes involved in photoreceptor homeostasis maintenance (**C**), the genes expressed in the photoreceptor IS, OS and synapses (**D**) and the genes important for phototransduction (**E**). Relative fold change was plotted against NLE. Data were expressed as mean ± SEM (n = 6 per group). **Compared to NLE, *P* < 0.01; ***compared to NLE, *P* < 0.001; ^#^compared to LE, *P* < 0.05; ^##^compared to LE, *P* < 0.01; ^###^compared to LE, *P* < 0.001. *HYP* the light-exposed mice treated with 50 mg/kg hyperoside, *LE* the light-exposed mice treated with vehicle, *NLE* the vehicle-treated mice without light exposure

As unveiled by the RNA-seq analyses summarized above (Fig. 7E), hyperoside treatment offset photooxidative stress-induced downregulation in multiple molecular pathways implicated in photoreceptor morphological and functional integrity (Fig. 8A). To further validate these findings, the retinas were collected from an independently repeated set of experiments and subjected to real-time qPCR analyses of the expression of transcription factors involved in photoreceptor gene expression, differentiation and functional development such as *Crx*, *Nr2e3* and *Nrl* (Fig. 8B); genes functioning in maintaining photoreceptor homeostasis such as *Abca4* and *Rdh12* (Fig. 8C); genes essential for photoreceptor structural components, including those required for connecting cilium organization such as *Bbs9*, *Rp1l1*, *Rpgrip1* and *Ush2a*, genes expressed predominantly in the photoreceptor IS and synapses, such as *Reep6* and genes expressed in the photoreceptor OS such as *Nxnl1*, *Prph2* and *Rom1* (Fig. 8D); genes critical for phototransduction, including *Cnga1*, *Gnat1*, *Guca1a*, *Opn1mw*, *Opn1sw*, *Pde6b*, *Rho* and *Slc24a1* (Fig. 8E). Consistent with the findings from the RNA-seq analyses (Figs. 7D, E and 8A), significantly reduced expression of the above-mentioned genes was observed in the LE retinas compared to the NLE retinas. However, the retinal expression of these genes was markedly increased in the HYP retinas compared to the LE retinas (Fig. 8B–E).

**Fig. 9:**
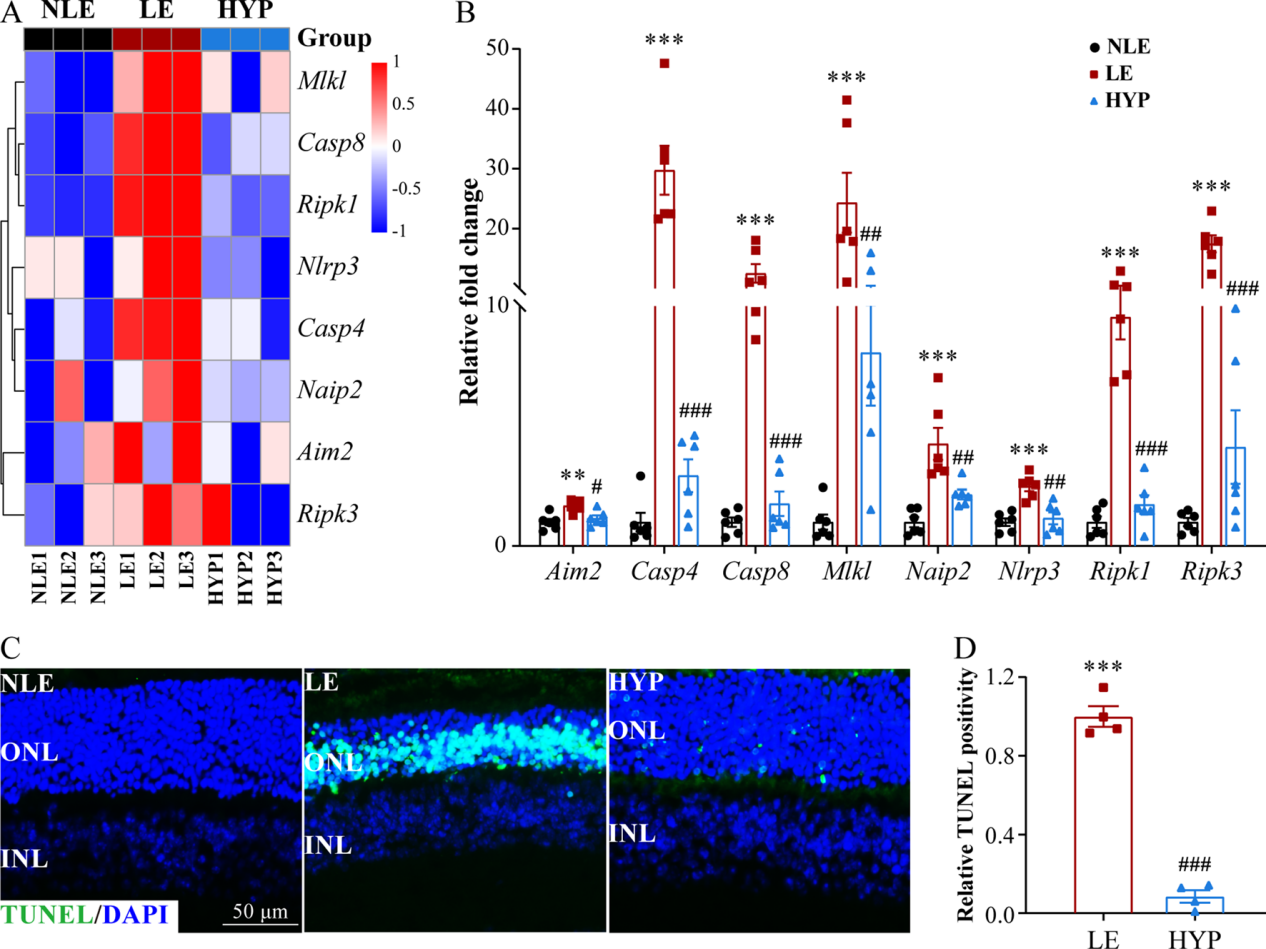
Hyperoside counteracts light-induced upregulation in the retinal expression of cell death regulators and mitigates photoreceptor cell death. **A** Heatmap visualization of the representative genes involved in programmed cell death revealed by RNA-seq analyses. **B** Real-time qPCR validation of the altered expression of the genes involved in apoptosis, necroptosis and pyroptosis. Relative fold change was plotted against NLE. Data were expressed as mean ± SEM (n = 6 per group). **C** TUNEL (in green) positivity in the retinas 3d post light exposure. DAPI (in blue) counterstaining marked the nuclei. Scale bar, 50 μm. **D** Relative fold change in the TUNEL positivity was plotted against LE. Data were expressed as mean ± SEM (n = 4 per group). **Compared to NLE, *P* < 0. 01; ***compared to NLE, *P* < 0.001; ^#^compared to LE, *P* < 0.05; ^##^compared to LE, *P* < 0.01; ^###^compared to LE, *P* < 0.001. *HYP* the light-exposed mice treated with 50 mg/kg hyperoside, *INL* inner nuclear layer, *LE* the light-exposed mice treated with vehicle, *NLE* the vehicle-treated mice without light exposure, *ONL* outer nuclear layer

In addition, RNA sequencing analyses revealed that hyperoside treatment mitigated photooxidative stress-induced upregulation of multiple pathways implicated in the regulation of programed cell death including apoptosis, necroptosis and pyroptosis (Fig. 7D, E). We therefore further validated the retinal expression of the gene signatures of these cell death pathways. Consistent with the results from RNA-seq analyses (Fig. 9A), compared to the NLE retinas, the LE retinas were marked by significantly elevated expression of *Casp8*, an essential player in the apoptosis pathway. In addition, significantly higher expression of necroptosis regulators such as *Mlkl*, *Ripk1* and *Ripk3* as well as pro-pyroptosis genes such as *Aim2*, *Casp4*, *Naip2* and *Nlrp3* was observed in the LE retinas. In contrast, the expression of these cell death regulators was remarkably decreased in the HYP retinas compared to the LE retinas (Fig. 9B). Further in situ assessment of photoreceptor cell death verified the findings from the gene expression analyses. As shown in Fig. 9C, prominent TUNEL positivity indictive of cell death events was detected in the ONL in the LE retinas. In stark contrast, much less TUNEL positivity was detected in the ONL in the HYP retinas (Fig. 9C, D).

Taken together, these results demonstrate that hyperoside treatment offset the deleterious impact of photooxidative stress on photoreceptor integrity and survival.

### Hyperoside-conferred photoreceptor protection is associated with attenuation of microglial activation and Müller cell gliosis in the retina

**Fig. 10:**
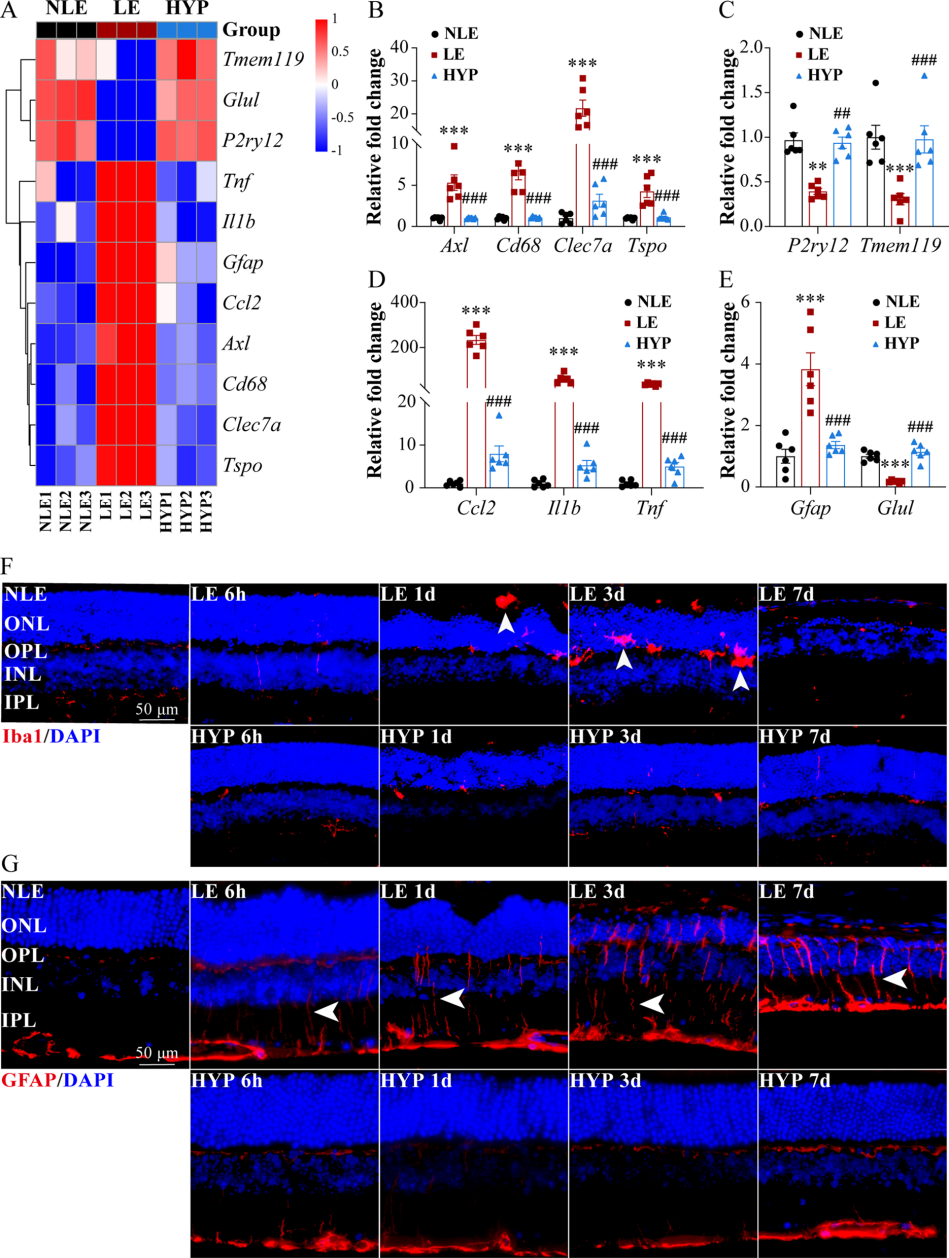
Hyperoside mitigates microglial inflammatory activation and Müller cell gliosis. **A** Heatmap visualization of the representative genes implicated in microglial inflammatory activation and Müller cell gliosis revealed by RNA-seq analyses. **B**–**E** Real-time qPCR analysis validation of the genes associated with disease-associated microglial activation phenotypes (**B**) and microglial homeostasis (**C**), inflammatory responses (**D**) and Müller gliosis (**E**). Relative fold change was normalized against NLE. **F** Iba1 immunopositivity (in red) in the retina. DAPI (in blue) was counterstained to label the nuclei. White arrowheads indicated activated microglia in the outer retina. Scale bar, 50 μm. **G** GFAP immunopositivity (in red) in the retina. DAPI (in blue) was counterstained to label the nuclei. White arrowheads indicated aberrant GFAP expression indicative of reactive gliosis. Scale bar, 50 μm. Data were expressed as mean ± SEM (n = 6 per group). **Compared to NLE, *P* < 0.01; ***compared to NLE, *P* < 0.001; ^##^compared to LE, *P* < 0.01; ^###^compared to LE, P < 0.001. *HYP* the light-exposed mice treated with 50 mg/kg hyperoside, *INL* inner nuclear layer, *IPL* inner plexiform layer, *LE* the light-exposed mice treated with vehicle, *NLE* the vehicle-treated mice without light exposure, *ONL* outer nuclear layer, *OPL* outer plexiform layer

In addition to degenerative pathologies manifested by photoreceptors per se, glial cells responsible for the maintenance of retina homeostasis also undergo reactive changes during the course of photoreceptor degeneration. It is known that photoreceptor degeneration is accompanied by neuroinflammation, a hallmark event signifying disturbed retinal homeostasis [18]. Photoreceptor degeneration-associated neuroinflammation is not only driven by activated microglia in the retina, but also under the tight regulation of Müller cells, a type of macroglia uniquely present in the retina [10, 19]. In keeping with this notion, our RNA-seq analyses revealed dysregulation in multiple pathways associated with inflammatory responses and microglial activation in the LE retinas, whereas hyperoside treatment counteracted photooxidative stress-triggered dysregulation of these pathways (Fig. 7D, E). A closer examination of the RNA-seq data unveiled that the signature genes associated with inflammatory responses and disease-associated microglial activation phenotypes (e.g. *Axl*, *Ccl2*, *Cd68*, *Clec7a*, *Il1b*, *P2ry12*, *Tmem119*, *Tnf* and *Tspo*) exhibited altered expression in the LE retinas. Meanwhile, altered expression of *Gfap* and *Glul* was also noted in the LE retinas, indicating reactive gliosis of Müller cells. However, hyperoside treatment counteracted the deleterious impact of the experimental light exposure on the retinal expression of these genes (Fig. 10A). Real-time qPCR analyses further validated the results from the RNA-seq analyses (Fig. 10B–E). IHC was also performed to visualize the changes in the microglia and Müller cells in situ. As shown in Fig. 10F, Iba1 positive microglia were readily detected in the photoreceptor ONL and subretinal space in the LE retinas 1d and 3d post illumination, spanning the time frame prior to the complete loss of photoreceptors noted 7d after light exposure. Whereas much less ectopic Iba1 immunopositivity was observed in the HYP retinas at the corresponding time points. The expression pattern of Gfap in the retina was also examined by IHC. The results revealed that Gfap immunopositivity was widely distributed across the retina in the LE retinas throughout the course of photoreceptor degeneration. In contrast, the expression pattern of Gfap in the HYP retinas were similar to that from the NLE retinas, remaining restricted primarily to the nerve fiber layer (Fig. 10G). Collectively, these results indicate that hyperoside treatment mitigates photooxidative stress-triggered neuroinflammatory responses and reactive activation in microglia and Müller cells in the retina.

## Discussion

In the present study, we demonstrate that hyperoside is pharmacologically active at protecting against oxidative stress-induced mitochondrial impairment and photoreceptor cell death in vitro. Most importantly, these cell-intrinsic photoreceptor protective activities of hyperoside are translatable in vivo as hyperoside attenuates photooxidative stress-induced photoreceptor degeneration on molecular, cellular, structural and functional levels. Moreover, hyperoside treatment counteracts neuroinflammatory responses and reactive alterations in microglia and Müller cells in the retina, providing additional evidence supporting the pharmacological significance of hyperoside in maintaining retina homeostasis under photooxidative stress conditions.

Our major findings here relate to the pharmacological implications of hyperoside in suppressing oxidative stress-mediated photoreceptor degeneration. Oxidative stress plays a pivotal role in triggering cell death, which is especially relevant in the case of photoreceptor cell death, the key cellular mechanism underlying photoreceptor degeneration [20]. This is due to highly specialized function of photoreceptors for light sensing, a process naturally generating ROS; high density of mitochondria, the primary source of ROS, in the photoreceptor IS; enrichment of the polyunsaturated fatty acids in the photoreceptor OS, rendering photoreceptors enhanced susceptibility to oxidation damage; anatomical adjacency of photoreceptors to the choroidal vasculature, an environment with high oxygen flow. Mitochondrial impairment is at the center of oxidative stress in that mitochondria are not only the major source of ROS, they are also the victims of the deleterious impact of ROS. Mitochondrial impairment and oxidative stress are early changes associated with photoreceptor degeneration [5]. Dysfunctional mitochondria further induce and aggravate oxidative stress [21, 22]. This mitochondria-based vicious cycle of overproduction of ROS is especially relevant for the pathophysiology of photoreceptor degeneration given that as high energy-consuming cells, photoreceptors contain approximately 90% of retina’s mitochondria and rely heavily on mitochondria for their survival and functionality. The critical role of intact mitochondria for photoreceptor health is also supported by the findings that photoreceptor degeneration is one of the major phenotypes of mitochondrial disorders [23, 24]. Therefore, protecting against mitochondrial impairment and oxidative stress serves as a viable route for the control of photoreceptor degeneration. Here we demonstrate that hyperoside suppresses oxidative stress-triggered mitochondrial impairment and photoreceptor cell death in vitro and in vivo. Hyperoside has been shown to directly counteract the deleterious impact of oxidative stress on the survival of PC12 neuronal cells [25]. Meanwhile, hyperoside mitigates Aβ-induced mitochondrial dysfunction in HT22 neuronal cells. The work here further demonstrates that hyperoside attenuates oxidative stress-induced mitochondrial dysfunction and cell death in 661W photoreceptor cells. Most importantly, our in vivo findings provide direct support to the significant effects of hyperoside on preserving the integrity of mitochondria under photooxidative stress conditions. Furthermore, hyperoside protects against photooxidative stress-induced loss of photoreceptor integrity on molecular, morphological, structural and functional levels. Putting these findings together, it is possible that hyperoside may exert photoreceptor protection in part by alleviating oxidative stress-induced mitochondrial impairment in photoreceptors. However, the molecular targets mediating the suppressive effects of hyperoside on oxidative stress-induced photoreceptor mitochondrial impairment and cell death remain to be identified in the future studies.

Aside from photoreceptors, it is worth noting that hyperoside treatment attenuates photoreceptor degeneration-associated neuroinflammatory responses and microglial activation in the retina. Retina is a complex neuronal tissue consisting of various types of neurons to execute the essential function of vision formation as well as non-neuronal cells that provide a supportive microenvironment underpinning the normal structural and functional homeostasis of the retina. Among the non-neuronal cellular constituents of the retina, glial cells, namely microglia, astrocytes and Müller cells, are equipped with important functions for maintaining a homeostatic microenvironment in the retina [26]. The pathophysiological implications of microglia in photoreceptor degeneration have been increasingly acknowledged. Under normal conditions, the resident immune cells of the retina, microglia, play an important role in maintaining retinal homeostasis via immune surveillance. Disturbance in the retinal microenvironment leads to rapid activation of microglia. More than merely an immediate response to the insults to the retina, aberrantly activated microglia are key players in mediating neuroinflammatory responses that are toxic to the retinal neurons, thereby exacerbating photoreceptor degeneration [10]. The results from our whole-genome gene expression profiling analyses demonstrate that LE retinas are characterized by significant upregulation of multiple molecular pathways associated with neuroinflammation and microglial activation. Most importantly, hyperoside treatment results in remarkable downregulation of the pathways implicated in neuroinflammation and microglial activation. In addition, microglia are normally located in the inner plexiform layer and outer plexiform layer in the retina. During photoreceptor degeneration, ectopic microglia are found in the outer nuclear layer and the subretinal space. Our results shown that hyperoside treatment also results in fewer microglia in the outer retina. Although it is likely that dampened activation of microglia is due to attenuated photoreceptor degeneration resulting from hyperoside treatment, a direct impact of hyperoside on microglial inflammatory activation is still possible. This possibility is supported by the findings that hyperoside suppresses lipopolysaccharide-induced inflammatory responses in microglia and attenuates the neurotoxic effects of activated microglia [27, 28]. Therefore, future studies are necessary to elucidate the potential implications of hyperoside in suppressing photoreceptor degeneration-associated microglia activation.

In addition to the impact on microglia, the suppressive effects of hyperoside on the reactive gliotic pathologies of Müller cells are also worth noting. Müller cells are not only the predominant glia of the retina, they are also the only glia specifically found in the retina. Müller cells closely interact with the retinal neurons including photoreceptors to maintain a homeostatic environment by regulating the metabolism as well as the extracellular milieu essential for the survival and normal function of the retinal neurons. Nearly all known retinal disorders are associated with a reactive gliotic changes in Müller cells, which is characterized by aberrantly upregulated expression of Gfap [11]. Although reactive gliosis of Müller cells is initiated with the intention to restore retinal homeostasis, it may accelerate the progression of retinal degeneration when Müller cells acquire malfunctional phenotypes defined in part by downregulation of the key enzyme glutamine synthetase encoded by *Glul*. Glutamine synthetase is primarily responsible for catalyzing the conversion of glutamate, a neurotransmitter that mediates normal excitatory synaptic transmission, to glutamine, the precursor of glutamate, thereby playing an essential role in regulating the glutamate-glutamine cycle, the shuttle of glutamate and glutamine between neurons and Müller cells. Inhibition of the glutamine synthetase leads to disturbed glutamate–glutamine cycle, causing marked reduction in the function of the retinal neurons including photoreceptors [29]. Injured photoreceptors release massive amounts of glutamate and excessive glutamate is neurotoxic. Downregulation of *Glul* expression can therefore directly cause insufficient metabolism of glutamate released by damaged photoreceptors, further exacerbating photoreceptor cell death. On the other hand, increasing the expression of glutamine synthetase confers protection against retinal degeneration [30]. Thus, gliotic Müller cells may fail to carry out their neuron-supportive functions and contribute to neuronal dysfunction and cell death [31]. Our findings here demonstrate that LE retinas are characterized by upregulated expression of *Gfap* and simultaneous downregulation of *Glul*. Meanwhile, aberrant expression pattern of Gfap is notable throughout the course of photoreceptor degeneration. These observations indicate that reactive gliosis of Müller cells is not only a phenotype of disturbed retinal homeostasis, it is also actively involved in the progression of photoreceptor degeneration under photooxidative stress conditions. Hyperoside treatment results in decreased expression of *Gfap* and increased expression of *Glul* in the light-exposed retinas. Meanwhile, the expression pattern of Gfap in the HYP retinas remains comparable to the NLE retinas. These results further highlight the beneficial impact of hyperoside on the retinal homeostasis under photooxidative stress conditions. However, whether hyperoside exerts direct impact on the pathophysiological changes in Müller cells remains to be investigated.

## Conclusions

The current work sheds new light on the pharmacological mechanisms of hyperoside in photoreceptor protection. Multiple levels of evidence presented here support the notion that hyperoside is pharmacologically effective at protecting against oxidative stress-mediated photoreceptor degeneration in part by suppressing oxidative stress-induced mitochondrial impairment and photoreceptor cell death. In addition, our findings here unveil that hyperoside treatment mitigates reactive gliosis in microglia and Müller cells in the retina, supporting the beneficial effects of hyperoside on maintaining retinal homeostasis under photooxidative stress conditions.

## Data Availability

Data will be made available by the corresponding author upon reasonable request.

## Abbreviations

DEGs: Differentially expressed genes
DMEM: Dulbecco’s modified Eagle’s medium
ERG: Electroretinography
FBS: Fetal bovine serum
GFAP: Anti-glial fibrillary acidic protein
GSEA: Gene set enrichment analysis
HE: Hematoxylin and eosin
IHC: Immunohistochemistry
INL: Inner nuclear layer
IS: Inner segment
MMP: Mitochondrial membrane potential
MPTP: Mitochondrial permeability transition pore
NES: Normalized enrichment score
OCT: Optical coherence tomography
ONL: Outer nuclear layer
OPL: Outer plexiform layer
OS: Outer segment
PCA: Principal component analysis
ROS: Reactive oxygen species
RPE: Retinal pigment epithelium
SEM: Standard error of mean
TEM: Transmission electron microscopy
TUNEL: Terminal deoxynucleotidyl transferase-mediated dUTP nick-end labeling

## References

[1] Hussey KA, Hadyniak SE, Johnston RJ (2022). Patterning and development of photoreceptors in the human retina. Front Cell Dev Biol.

[2] Wright AF, Chakarova CF, Abd El-Aziz MM, Bhattacharya SS (2010). Photoreceptor degeneration: genetic and mechanistic dissection of a complex trait. Nat Rev Genet.

[3] Pardue MT, Allen RS (2018). Neuroprotective strategies for retinal disease. Prog Retin Eye Res.

[4] Moos WH, Faller DV, Glavas IP, Harpp DN, Kamperi N, Kanara I, Kodukula K, Mavrakis AN, Pernokas J, Pernokas M (2022). Treatment and prevention of pathological mitochondrial dysfunction in retinal degeneration and in photoreceptor injury. Biochem Pharmacol.

[5] Vlachantoni D, Bramall AN, Murphy MP, Taylor RW, Shu X, Tulloch B, Van Veen T, Turnbull DM, McInnes RR, Wright AF (2011). Evidence of severe mitochondrial oxidative stress and a protective effect of low oxygen in mouse models of inherited photoreceptor degeneration. Hum Mol Genet.

[6] Baksheeva VE, Tiulina VV, Tikhomirova NK, Gancharova OS, Komarov SV, Philippov PP, Zamyatnin AA, Senin II, Zernii EY (2018). Suppression of light-induced oxidative stress in the retina by mitochondria-targeted antioxidant. Antioxidants (Basel).

[7] Atanasov AG, Zotchev SB, Dirsch VM, Supuran CT (2021). Natural products in drug discovery: advances and opportunities. Nat Rev Drug Discov.

[8] Wu H, Zhu B, Li D, Xu J, Chang J, Du X, Cui J, Zhang N, Zhang T, Chen Y (2022). *Cuscuta chinensis* Lam. protects against light-induced retinal degeneration: therapeutic implications for photoreceptor degenerative disorders. Front Pharmacol.

[9] Zeng KW, Wang XM, Ko H, Kwon HC, Cha JW, Yang HO (2011). Hyperoside protects primary rat cortical neurons from neurotoxicity induced by amyloid β-protein via the PI3K/Akt/Bad/Bcl(XL)-regulated mitochondrial apoptotic pathway. Eur J Pharmacol.

[10] Rashid K, Akhtar-Schaefer I, Langmann T (2019). Microglia in retinal degeneration. Front Immunol.

[11] Bringmann A, Pannicke T, Grosche J, Francke M, Wiedemann P, Skatchkov SN, Osborne NN, Reichenbach A (2006). Müller cells in the healthy and diseased retina. Prog Retin Eye Res.

[12] Al-Ubaidi MR, Font RL, Quiambao AB, Keener MJ, Liou GI, Overbeek PA, Baehr W (1992). Bilateral retinal and brain tumors in transgenic mice expressing simian virus 40 large T antigen under control of the human interphotoreceptor retinoid-binding protein promoter. J Cell Biol.

[13] Sivandzade F, Bhalerao A, Cucullo L (2019). Analysis of the mitochondrial membrane potential using the cationic JC-1 dye as a sensitive fluorescent probe. Bio Protoc.

[14] Wu H, Xu J, Du X, Cui J, Zhang T, Chen Y (2020). Shihu Yeguang Pill protects against bright light-induced photoreceptor degeneration in part through suppressing photoreceptor apoptosis. Biomed Pharmacother.

[15] Bolger AM, Lohse M, Usadel B (2014). Trimmomatic: a flexible trimmer for Illumina sequence data. Bioinformatics.

[16] Trapnell C, Williams BA, Pertea G, Mortazavi A, Kwan G, van Baren MJ, Salzberg SL, Wold BJ, Pachter L (2010). Transcript assembly and quantification by RNA-Seq reveals unannotated transcripts and isoform switching during cell differentiation. Nat Biotechnol.

[17] Anders S, Pyl PT, Huber W (2015). HTSeq—a Python framework to work with high-throughput sequencing data. Bioinformatics.

[18] Kaur G, Singh NK (2021). The role of inflammation in retinal neurodegeneration and degenerative diseases. Int J Mol Sci.

[19] Chen Y, Xia Q, Zeng Y, Zhang Y, Zhang M (2022). Regulations of retinal inflammation: focusing on Müller glia. Front Cell Dev Biol.

[20] Wenzel A, Grimm C, Samardzija M, Remé CE (2005). Molecular mechanisms of light-induced photoreceptor apoptosis and neuroprotection for retinal degeneration. Prog Retin Eye Res.

[21] Guo C, Sun L, Chen X, Zhang D (2013). Oxidative stress, mitochondrial damage and neurodegenerative diseases. Neural Regen Res.

[22] Kowalczyk P, Sulejczak D, Kleczkowska P, Bukowska-Ośko I, Kucia M, Popiel M, Wietrak E, Kramkowski K, Wrzosek K, Kaczyńska K (2021). Mitochondrial oxidative stress—a causative factor and therapeutic target in many diseases. Int J Mol Sci.

[23] Zeviani M, Carelli V (2021). Mitochondrial retinopathies. Int J Mol Sci.

[24] McKechnie NM, King M, Lee WR (1985). Retinal pathology in the Kearns-Sayre syndrome. Br J Ophthalmol.

[25] Liu Z, Tao X, Zhang C, Lu Y, Wei D (2005). Protective effects of hyperoside (quercetin-3-o-galactoside) to PC12 cells against cytotoxicity induced by hydrogen peroxide and tert-butyl hydroperoxide. Biomed Pharmacother.

[26] Reichenbach A, Bringmann A (2020). Glia of the human retina. Glia.

[27] Fan HH, Zhu LB, Li T, Zhu H, Wang YN, Ren XL, Hu BL, Huang CP, Zhu JH, Zhang X (2017). Hyperoside inhibits lipopolysaccharide-induced inflammatory responses in microglial cells via p38 and NFκB pathways. Int Immunopharmacol.

[28] Wang K, Lu C, Wang T, Qiao C, Lu L, Wu D, Lu M, Chen R, Fan L, Tang J (2022). Hyperoside suppresses NLRP3 inflammasome in Parkinson’s disease via pituitary adenylate cyclase-activating polypeptide. Neurochem Int.

[29] Barnett NL, Pow DV, Robinson SR (2000). Inhibition of Müller cell glutamine synthetase rapidly impairs the retinal response to light. Glia.

[30] Gorovits R, Avidan N, Avisar N, Shaked I, Vardimon L (1997). Glutamine synthetase protects against neuronal degeneration in injured retinal tissue. Proc Natl Acad Sci USA.

[31] Bringmann A, Grosche A, Pannicke T, Reichenbach A (2013). GABA and glutamate uptake and metabolism in retinal glial (Müller) cells. Front Endocrinol (Lausanne).

